# Secondary stressors and extreme events and disasters: a systematic review of primary research from 2010-2011

**DOI:** 10.1371/currents.dis.a9b76fed1b2dd5c5bfcfc13c87a2f24f

**Published:** 2012-10-29

**Authors:** Sarah Lock, G. James Rubin, Virginia Murray, M. Brooke Rogers, Richard Amlôt, Richard Williams

**Affiliations:** Centre for Radiation, Chemical and Environmental Hazards (CRCE) and Extreme Events; King's College London; Health Protection Agency; King's College London; Health Protection Agency; University of Glamorgan

## Abstract

Introduction Extreme events and disasters, such as earthquakes and floods, cause distress and are associated with some people developing mental disorders. Primary stressors inherent in many disasters can include injuries sustained or watching someone die. The literature recognises the distress which primary stressors cause and their association with mental disorders. Secondary stressors such as a lack of financial assistance, the gruelling process of submitting an insurance claim, parents’ worries about their children, and continued lack of infrastructure can manifest their effects shortly after a disaster and persist for extended periods of time. Secondary stressors, and their roles in affecting people’s longer-term mental health, should not be overlooked. We draw attention in this review to the nature of secondary stressors that are commonly identified in the literature, assess how they are measured, and develop a typology of these stressors that often affect people after extreme events. Methods We searched for relevant papers from 2010 and 2011 using MEDLINE®, Embase and PsycINFO®. We selected primary research papers that evaluated the associations between secondary stressors and distress or mental disorders following extreme events, and were published in English. We extracted information on which secondary stressors were assessed, and used thematic analysis to group the secondary stressors into a typology. Results Thirty-two relevant articles published in 2010 and 2011 were identified. Many secondary stressors were poorly defined and difficult to differentiate from primary stressors or other life events. We identified 11 categories of secondary stressors, though some extend over more than one category. The categories include: economic stressors such as problems with compensation, recovery of and rebuilding homes; loss of physical possessions and resources; health-related stressors; stress relating to education and schooling; stress arising from media reporting; family and social stressors; stress arising from loss of leisure and recreation; and stress related to changes in people’s views of the world or themselves. Limitations in this review include its focus on studies published in 2010 and 2011, which may have led to some secondary stressors being excluded. Assumptions have been made about whether certain items are secondary stressors, if unclear definitions made it difficult to differentiate them from primary stressors. Conclusions This is the first review, to our knowledge, that has developed a typology of secondary stressors that occur following extreme events. We discuss the differing natures of these stressors and the criteria that should be used to differentiate primary and secondary stressors. Some secondary stressors, for example, are entities in themselves, while others are persisting primary stressors that exert their effects through failure of societal responses to disasters to mitigate their immediate impacts. Future research should aim to define secondary stressors and investigate the interactions between stressors. This is essential if we are to identify which secondary stressors are amenable to interventions which might reduce their impacts on the psychosocial resilience and mental health of people who are affected by disasters. Corresponding Author: Dr Sarah Lock, Extreme Events and Health Protection, London, 151 Buckingham Palace Road, London, SW1W 9SZ. E-mail: sarah.lock@hpa.org.uk

## Introduction

Rapid and extensive changes occur in people’s lives and the worlds in which they live when they are exposed to extreme events and disasters. These can cause great stress to people, families and communities because of their inherent effects, such as causing short-term fear of death and exposure to traumatic events, and because of the chain of events they set in motion. Effective psychosocial recovery after disasters and other extreme events is characterised by adaptation to changed circumstances. In some situations, the personal and social meanings that people derive from their experiences of an extreme event have more influence on its psychosocial impact than the event itself. People’s abilities to re-build, recover, and adapt following a disaster are determined by their own physical, psychological and social characteristics, as well as the characteristics of, and support they receive from their families and the communities in which they live 1.

The Department of Health in England (DH) 2 and NATO/EAPC 3 clearly differentiate between distress and mental disorders following a disaster. Most people experience distress after their exposure to an extreme event. In the case of people with good psychosocial resilience and access to social support, their distress may be relatively transient as people call on a set of inner capabilities and supporting relationships to spring back and begin the processes of adaptation. Mental disorders occur often, but less commonly than distress, and in some cases they may require intensive and long term continuing interventions and treatment. The threshold between what might be considered a common or anticipated response of distress, and what is indicative of a person developing a mental disorder, are difficult to define and the pathways are complex. There are a number of factors that make it more likely for people to develop mental disorders such as pre-existing mental ill health and personal factors such as gender, age or income, but particularly important factors are whether or not they receive adequate social support and whether or not they perceive that they receive this. As the timescale and dysfunctional impact of distress grows, the possibility of people passing from an anticipated, distressed psychosocial response, to developing mental disorder also increases 3.

Furthermore, the psychosocial effects of extreme events have commonly been viewed as resulting from a complex array of primary and secondary stressors. Primary stressors have been defined as stress that is *‘inherent in particular major incidents, disasters and emergencies and arising directly from those events*’ 2. They encompass experiences that are related directly to, or consequent on, people’s involvement in a disaster such as watching someone being killed, or fearing for one’s life and the safety of others.

Secondary stressors, by contrast, are circumstances, events or policies that are indirectly related or ‘*non-inherent and consequential’* 2 to the index extreme event. Typically, the term is used to describe situations that persist for longer than the events. Some secondary stressors are entities in themselves, whereas others are unresolved and persisting primary stressors. They can include problems such as infrastructure failure and challenges to returning to normality and repairing structures. They may also include the impacts of policies and plans made prior to events that inadvertently limit people’s recovery or adaptation.

Much of the focus of previous research has been to identify primary stressors and provide evidence and strategies to tackle these problems and minimise their impact 2 ^,^ 3. However, recent initiatives have resulted in increasing awareness of the importance of secondary stressors and their potential to lengthen the impact and distress caused by a disaster 2 ^,^ 3 ^,^ 4. There is plentiful evidence that the likelihood of people developing distress or going on to develop a mental disorder is increased in line with the intensity and persistence of both primary and secondary stressors. Despite this, however, there is a lack of a clear typology to describe these secondary stressors. We believe that it is important to be able to group the secondary stressors that occur after an extreme event. This should enable improved application of past experience with a view to recognising potentially important secondary stressors earlier, and, preferably, even before a disaster occurs.

In this review we aimed to: determine if secondary stressors that affect people exposed to extreme events and disasters are commonly recognised in the literature; identify how they are measured; and produce a typology of secondary stressors. In particular, we sought to identify the secondary stressors that contribute to prolonging or intensifying people’s distress and those which may also increase the risks of survivors developing mental disorders following extreme events.

## Methods

We identified and analysed the content of primary research papers. We limited our search to research papers published in 2010 and 2011 because the disaster literature has grown rapidly in the last few years. Our intention is to use analysis of these papers to provide an indication of how secondary stressors are described in the current literature.

In order to be included in our review, papers had to report an evaluation of the association between a secondary stressor and an impact on people’s distress or mental health after an extreme event. Initially, we defined secondary stressors as ‘*continuing or chronic problems that occur as a consequence of a disaster and impact on people’s emotional, cognitive, social and physical functioning; and thereby may prolong the impact of the event. They are not directly related to or inherent in the event’.*

We conducted a free text literature search using MEDLINE^®^, Embase and PsycINFO^®^ for potentially relevant papers published in English. First, we assembled a list of potential secondary stressors. This was used to compile a list of search terms to use alongside the generic term ‘*secondary stressor*’. We also used the WHO Global Platform for disaster risk reduction list of definitions^5^ to define the types of extreme event that we included in the search terms. No grey literature or citations searches were performed and data extraction was restricted to the information presented in the published papers.

The keywords and search strategy used was: second* stress* OR econom* OR cost* OR income* OR employ* OR unemploy* OR insur* OR compensat* OR legal* OR propert* OR hous* OR business* OR shelter* OR displace* OR refuge* OR evacuat* OR relation* OR marit* OR famil* OR social* OR role* OR fear recurr* OR welf* OR affluenc* OR stigma*OR distrust* OR mistrust* AND extreme event* OR cyclone* OR typhoon* OR hurricane* OR tornado* OR tropic* storm* OR heatwave* OR heat-wave* OR heat wave* OR flood* OR drought* OR earthquake* OR volcan* erupt* OR volcan* ash* OR landslide* OR tsunami* OR wildfire* AND mental* OR distress*, limited to English Language.

One author (SL) conducted the search, evaluated papers against our inclusion criteria, and extracted data from the papers. A quality review of articles was not performed as the primary aim of this study was to identify the types of secondary stressors that have most recently been studied in the literature, regardless of the quality of those studies. Key details extracted from the full texts included: study design; population; country; type of extreme event; secondary stressor types, how and when they were measured after the extreme event; mental health outcomes. Where papers reported that their authors had studied secondary stressors as a group, data on each separate factor in the group were extracted.

We then used thematic analysis to group the secondary stressors into a typology. This grouping was problematic when reviewing papers in which the single generic term of ‘*secondary stressors*’ was used to describe many different problems following disasters. Analysis of one paper, for example, revealed that the secondary stressors measured after a hurricane included eight separate items, including impacts on housing, family, friendships and employment 6. In these instances, we considered each item separately for our thematic analysis.

## Results

We identified and reviewed the abstracts of 257 papers published in 2010 or 2011. We could not obtain the full texts of four articles. Forty-two papers seemed potentially relevant to our review and we read and analysed the full texts. Thirty-two papers fulfilled our inclusion criteria 6 ^-^ 37.

**General findings**

The papers reported research on a range of extreme events in a variety of countries (Table 1). The majority of papers reported on the impacts on the mental health of adults 7 ^,^ 8 ^,^ 10 ^,^ 11 ^,^ 12 ^,^ 13 ^,^ 14 ^,^ 16 ^,^ 18 ^,^ 20 ^,^ 24 ^,^ 25 ^,^ 26 ^,^ 27 ^,^ 30 ^,^ 31 ^,^ 32 ^,^ 34 ^,^ 35 ^,^ 37. Others focused on adults and adolescents above the age of 15 years 19 ^,^ 22 ^,^ 28, while nine reported research on children and adolescents aged between 4 and 17 years 6 ^,^ 9 ^,^ 15 ^,^ 17 ^,^ 21 ^,^ 23 ^,^ 29 ^,^ 33 ^,^ 36.

**Table 1: Extreme event by country:** 

Country	Extreme event
Australia	Cyclone^36^
China	Earthquakes 18 ^,^ 19 ^,^ 20 ^,^ 22 ^,^ 23 ^,^ 24 ^,^ 25; Flooding^33^
Greece	Wildfires 37
Indonesia	Earthquakes and Tsunamis 28
Pakistan	Earthquakes 26
South Korea	Flooding 32
Sri Lanka	Earthquakes and Tsunamis 27 ^,^ 29 ^,^ 30
Turkey	Earthquakes 21
UK	Flooding 31 ^,^ 34 ^,^ 35
USA	Hurricane 6 ^,^ 7 ^,^ 8 ^,^ 9 ^,^ 10 ^,^ 11 ^,^ 12 ^,^ 13 ^,^ 14 ^,^ 15 ^,^ 16 ^,^ 17

**Primary stressors, concurrent life events or secondary stressors**

Usually, the focus of the research reported in the papers we read related to identifying and minimising the impacts of primary stressors.

One key problem within the studies reviewed was the lack of a definition, or the use of unclear definitions, for many of the secondary stressors. We found that researchers assessed secondary stressors using different methods or tools. The time between the occurrence of the index extreme event and the measurement of secondary stressors ranged widely from as little as one month to as long as four years. These features made it difficult for us to determine if a problem was a primary stressor, concurrent life event, or a secondary stressor.

Another challenge arose due to the blurred definitions and time boundaries between primary and secondary stressors. Some authors reported research on items such as ‘*lost livelihood*’ 19 ^,^ 27 or property destruction 16 ^,^ 19 ^,^ 24 ^,^ 37. They measured these factors as part of ‘*event-related traumatic events*’ alongside other experiences such as seeing people die, fearing for one’s life at the time of the event, or the severity of the disaster. Arguably, these circumstances are aspects of the extreme event and should, therefore, be considered as being among the primary stressors that stem directly from the event. However, as clearer definitions or explanations of these terms were not provided, and as the studies were conducted up to three years after the index event, they could also be seen as unresolved primary stressors or secondary stressors.

Similarly, daily or continuing stressors and other ‘*negative life events*’ were measured in some research 7 ^,^ 9 ^,^ 14. These events included changes to marital or family status, death of a spouse or family member, divorce, marriage, other family and work problems. Once again, the definition of these terms, and the variations in time intervals after the extreme event at which they were measured, were not clear. These occurrences could be either a direct consequence of, or exacerbated by the disaster, or be concurrent and unrelated. We have not included these events within our typology of secondary stressors.

**A typology of secondary stressors**

**** Table 2 summarises the results of our thematic analysis of the secondary stressors.

Although this table suggests clear distinctions between the different categories of secondary stressors, many of the stressors fall into more than one category in the typology. Different stressors may also interact and or act in a cumulative fashion to prolong distress or raise the risk of people developing mental disorders. None of the studies examined the pathways or inter-relationships between the primary and secondary stressors, or between different secondary stressors.

**Table 2: A Typology of Secodary Stressors:** 

Category	Examples from the literature
Economic	Loss of continuing loss of income 11 ^,^ 12 ^,^ 16 ^,^ 30 ^,^ 31 ^,^ 34 Loss or lack of employment 6 ^,^ 10 ^,^ 11 ^,^ 16 ^,^ 19 ^,^ 22 ^,^ 27 ^,^ 28 Impact on house values 31
Difficulties with compensation	Lack of help, advice and information for applications to insurers and providers of grants or loans 30 ^,^ 34 ^,^ 35 Conflicting information 35 Applications for insurance payouts or state grants delayed or denied 16 ^,^ 32 ^,^ 34 ^,^ 35
Problems with recovery and rebuilding homes	Continuing lack of essential services 7 ^,^ 22 ^,^ 31 Lack of information or advice to understand the application process for rebuilding property 34 ^,^ 35 Progressive damage to houses 6 ^,^ 13 ^,^ 16 ^,^ 19 ^,^ 22 ^,^ 24 ^,^ 37 Continuing to live in temporary accommodation 16 ^,^ 18 ^,^ 20 ^,^ 22 Lack of housing assistance 10 Problems with restoration of homes or property 30 ^,^ 35 Dealing with daily life and recovery processes 14 ^,^ 28 ^,^ 35
Loss of physical possessions or resources	Loss of car, furniture, appliances and clothing, including items of sentimental value 11 ^,^ 13 ^,^ 19
Health	New or continuing health concerns or conditions 11 ^,^ 13 ^,^ 14 ^,^ 22 ^,^ 31 Lack of access to healthcare 10 ^,^ 22 Lack of access to psychosocial care 10 Lack of access to prescription medication 10
Education and Schooling	Lack of education opportunities or facilities 11 Loss of socialisation that is part of attending school 6 ^,^ 8 Changing to new schools or education establishments 6 ^,^ 8
The media	Exposure to negative media reports 23
Familial	Breakdown of relationships and loss of intimacy 11 ^,^ 12 ^,^ 14 ^,^ 22 ^,^ 26 ^,^ 28 ^,^ 29 ^,^ 31 Breakdown in household activities and functioning 11 ^,^ 14 ^,^ 15 ^,^ 17 ^,^ 28 ^,^ 36 Breakdown of familial resilience 30 ^,^ 36 Changes to household composition 6 Parental psychopathology 4 ^,^ 15 ^,^ 21 ^,^ 33 Impact on parenting skills 15 ^,^ 17 ^,^ 29 ^,^ 33 Physical and mental abuse and neglect of partners or children 15 ^,^ 17 ^,^ 29 ^,^ 33
Social	Physical separation from friends 6 Disruption to social networks and relationships 11 ^,^ 14 ^,^ 21 ^,^ 24 ^,^ 28 ^,^ 30 ^,^ 34 ^,^ 36 Reduction in level of social support 7 ^,^ 11 ^,^ 13 ^,^ 15 ^,^ 18 ^,^ 19 ^,^ 20 ^,^ 21 ^,^ 23
Leisure and recreation	Disruption to leisure and relaxation activities 11 ^,^ 14 ^,^ 28
Changes in the view of the world or oneself	Loss of control and agency 11 ^,^ 14 ^,^ 25 Loss of aspirations for the future 11 ^,^ 14 Fear of recurrence of an extreme event 22 ^,^ 23 ^,^ 35

**Economic Stressors.** A variety of continuing economic problems may act as secondary stressors following extreme events. They include the loss of income 11 ^,^ 16 ^,^ 22 following loss of employment, and the loss of savings, emergency money or retirement security 11 through having to pay for repairs or recovery. Lack of financial assistance or support to help to improve people’s economic situations 30 ^,^ 34 or loss of financial credit 11 may well contribute to continuing distress and anxiety. These effects have been identified as occurring from as soon as ten weeks to as long as 32 months after an event. A ‘*negative perceived impact on finances*’ increased the risk of people developing mental health problems up to six months after a flood in England 31.

Loss of employment *or ‘livelihood’* 6 ^,^ 10 ^,^ 16 ^,^ 19 ^,^ 22 ^,^ 27 ^,^ 28, lack of stable employment 11 or employment assistance 10, along with the loss of farmland 22, business 16 or tools required for work 11 can also contribute to distress and mental health problems. Parents’ persisting loss of a job was associated with their children developing the symptoms of PTSD 18 months after a hurricane 6.

A 'n*egative perceived impact on house values' *caused by a flood 31 was investigated as a potential secondary stressor. It was not found to have a significant impact on mental health outcomes in that study.

**Stress arising from Problems with Compensation.** Problems with applications for financial assistance or compensation through insurers or loss adjustors were highlighted as secondary stressors. People may lack understanding of application processes and the roles, responsibilities and rights of the various parties 34 ^,^ 35. Often, there is insufficient help or advice in making claims 30 ^,^ 34, and, at times, conflicting information is given to applicants 35 or problems arise from ‘*inconsiderate attitudes*’ 35.

Lack of compensation or insurance payments after an event may also contribute to psychological stress or mental disorders such as depression 16 ^,^ 35. Secondary stressors include disputes with insurance companies, state grant lenders, and loan providers over applications that were not processed,or were delayed or denied 16 ^,^ 35. There were reports of lack of satisfaction with insurance payouts 32 or refusals to re-insure properties in high-risk areas in the future 34. There were also problems with affected people being restricted in purchasing new possessions by the terms of their policies or by a lack of available money 35. These impacts on income and savings contribute further to future economic insecurity, especially in the event of people being affected by repeated disasters.

**Stress arising from Recovery and Rebuilding Homes.** Recovery and rebuilding processes start soon after disasters and may continue for many years. There are numerous secondary stressors that can contribute to people’s distress and mental health problems. Many people lack understanding, information or advice about recovery processes 34 ^,^ 35. There are reports of continuing lack of essential services such as gas, electricity and water supplies 7 ^,^ 31 and medical services 22, which may present difficulties.

Often, people who are affected have to contend with continued house damage 6 ^,^ 13 ^,^ 16 ^,^ 19 ^,^ 22 ^,^ 24 ^,^ 37 and some live in temporary accommodation such as caravans or dormitories 16 ^,^ 18 ^,^ 20 ^,^ 22. Other people experience lack of housing assistance 10. People who are restoring their homes can come up against difficulties with building contractors. They include delays in the work being done, ‘inconsiderate attitudes’, giving homeowners no control over rebuilding and poor workmanship 35. Lack of community participation or help in repairing and constructing homes can be a stressor for some people 30.

Impact on life in general 28, such as having to get on with one’s daily routine of work and managing one’s family while dealing with recovery and associated problems 14 ^,^ 35, are also reported as secondary stressors.

**Stress arising from Loss of Physical Possessions or Resources.** Many people experience the loss of their physical possessions and resources such as cars, furniture, home appliances and clothing 11 ^,^ 13 ^,^ 19. The impact of replacing these items can be substantial, especially if there are delays in payouts from insurance policies or grants, and the sentimental value of some lost or damaged items may make them irreplaceable.

**Health-related Stressors.** Health problems that have been measured in studies as potential secondary stressors include concerns about the health of self, family and friends 11 ^,^ 13 ^,^ 14 ^,^ 22 ^,^ 31. Often, we found it difficult to determine if papers were describing new health problems caused specifically by the index event, or a continuation or exacerbation of pre-existing illnesses.

Other problems include lack of access to medical care 10 ^,^ 22 or prescription medication 10. There are reports of people having problems in gaining access to psychological services such as counselling or therapy for carers, children or families 10 up to one or two years after an event.

**Stress relating to Education and Schooling.** The negative impacts of disasters on schooling, study and tutoring have been studied. Some studies looked at the negative impacts on mental health of going to a new school 6, being displaced from your ‘*home university*’ 8 and losing the opportunity for further education or training 11. However, two of the papers we identified provided no further explanation of these terms and the lack of detail made it difficult to assess what particular concepts these studies measured 10 ^,^ 28.

**Stress arising from Media Reporting.** Media coverage after disasters is often extensive. One study 23 found that being exposed to negative media reports of ‘*scary or sorrowful stories*’ one month after an earthquake increased the risk of depression and the demand for psychological counselling services.

**Family Stressors.** Changes to the functioning of families following disasters can be problematic, both by eroding the protective or buffering effects that social support has on the effects of adversity, and also by introducing new forms of upsetting experiences. Secondary stressors studied in families include breakdown in relationships, reduced social support from a partner 31, ‘*inter-parental conflict*’, intimate partner abuse 12 ^,^ 26 ^,^ 29, changes to marital status 14 and ‘*negative impacts on family relationships*’ 28. Reduced levels of intimacy can occur and they have been measured as ‘*negative impacts on sexual activity*’ 22 or a loss of time spent with loved ones 11 ^,^ 14.

Breakdown in household daily activities and functioning 11 ^,^ 14 ^,^ 15 ^,^ 17 ^,^ 28 ^,^ 36 that ordinarily provide stability and routine can also be a secondary stressor. Changes to the composition of households through loss of family members, or having additional family members or friends living with you for some time after an event 6, further disrupts family life and the perceptions that people have of their families as unified.

A lack of ‘*family resilience*’ has also been found to contribute to generating people’s perceptions of distress and mental disorders. The term ‘*resilience*’ was used in two papers to describe the ability of families to talk about worries and problems and for members of families to help each other in times of need and adversity 30 ^,^ 36. It was difficult to determine if a breakdown in resilience occurred as a result of the disaster impacting on otherwise functioning families and was, therefore, a secondary stressor, or if this was a pre-existing vulnerability.

A number of papers that we reviewed reported studies of the negative impacts of psychopathology (such as depression or PTSD) in parents on the emotional and mental health of their children. This can occur either directly 4 ^,^ 15 ^,^ 21 ^,^ 33 or through pathways such as poor parenting practice or verbal and physical abuse 15 ^,^ 17 ^,^ 29 ^,^ 33.

**Social Stressors.** Just as family support networks are important in helping people to cope with the stress of extreme events, so too are other social networks and social support. Disruption and breakdown of social networks and relationships with friends, work colleagues and the wider community all contribute as secondary stressors. These concepts have been reported in the literature as ‘*negative impacts on friendship*’ or loss of companionship 11 ^,^ 24 ^,^ 28, changes and disruptions to social networks 14 ^,^ 21 ^,^ 30 ^,^ 36 and lack of social capital 34. Physical separation from family and friends 6 remains an important stressor even up to 18 months after an event.

Reduced levels of social support, loyalty and mutuality from parents, peers, teachers and the wider community have been highlighted as matters that contribute to mental disorders 7 ^,^ 11 ^,^ 13 ^,^ 15 ^,^ 18 ^,^ 19 ^,^ 20 ^,^ 21 ^,^ 23. However, the definition of social support varies across the papers we reviewed. Some used validated tools to measure this concept, while others asked a single question or gave no further description of what this term encompassed.

**Stress arising from Loss of Leisure and Recreation.** Disruption to leisure and relaxation activities 28, lack of time for sleep 14 or of free time to spend with families and friends 11 can result from having to spend time and effort organising recovery and rebuilding. Pressure on time can contribute to any existing distress and mental health problems.

**Stress related to changes in the view of the world or oneself.** Commonly after a disaster, people may have a range of psychological experiences and they too, may act as secondary stressors and contribute to distress or mental disorders. Psychological problems that have been studied include the feeling of loss of control and agency. Commonly, people may experience feeling reduced levels of independence 11 ^,^ 14 or value to others 11 and feel that they ‘*lack control over their environment*’ or ‘*predictability over their future*’ 25. Some people lose their aspirations for the future. They lack goal setting 11 ^,^ 14, feel their lives have lost meaning or purpose 11 or they lack optimism about the future 14.

Fear of recurrence of the extreme event is another significant issue for many people 22 ^,^ 23 ^,^ 35 especially, for example, when they experience aftershocks following earthquakes or heavy rain after floods.

## Discussion

**Key findings**

Disasters can have major, long-term impacts on people, families and communities. Distress and mental disorders can be caused by the direct effects of the extreme event (primary stressors), and also by the knock-on effects of secondary stressors.

One of the key findings of our review is that, while secondary stressors are often reported in the literature, there appears to be no agreed definition of what constitutes a secondary stressor at least within the latest research. Many of the secondary stressors reported are also poorly described and are measured using different tools or after different time intervals. This hampered our efforts to develop a typology of secondary stressors.

Despite this, after our review, we suggest that 11 categories of secondary stressor should be considered in future studies. Some of them are pragmatic. They reflect the loss of physical resources, economic problems such as difficulties with compensation, problems with recovery and rebuilding, loss of physical possessions and resources, problems with health, education and schooling, and the impact of the media.

Other secondary stressors are more psychological or social in nature. They reflect the impact of a disaster on perceptions of self or the world around affected people, the ways in which families or social support networks interact, or the time available for recreation and leisure. All these problems are important in contributing to the distress and mental disorders that can afflict people who are affected by an extreme event. However, different approaches may be required to mitigate the stressors that are more objective, compared to those that appear less tangible. Doing this requires understanding of how secondary stressors exert their effect. This can be via a complex set of mechanisms.

**Mechanisms of interaction**

At one end of the spectrum, a primary stressor such as the death of a loved one can cause intense distress and grief for close friends or relatives. This can also intensify the experiences of people who went through the same ordeal and survived. While social support offered by families and other community groups has been shown to be vitally important in moderating the effects of disasters on people, relationships in families and other community groups may also create circumstances in which stress is communicated.

Lack of family support networks can contribute to parental psychopathology. In turn, this can affect adults’ parenting, which then contributes to their children’s distress. Thus, the impact of events on adults may cause secondary effects, or burden, to fall on their children at a time when their usual needs have been intensified by their experiences of the same disaster. Viewed from the children’s perspectives, the impact of events on them can increase the burden of care that falls on their parents at a time when they may be struggling to cope with their own experiences.

Reverberating networks of stress, such as these, can be set up because people worry about each other. That can render the primary impact on one person a source of secondary stress for others. It is easy to see that social support from families is vital. However, interaction between primary and secondary stressors within families and communities can also lead to mental disorders. These complex pathways of interaction, which are not unique to family-related stressors, were not studied in the research papers that we identified in this review. We suggest that empirical research into the mechanisms of interaction of stressors could contribute to development of more effective interventions.

Overall, the lack of clarity that we found in the literature makes the tasks of developing a typology of secondary stressors and examining the mechanisms by which different sources of stress interact particularly challenging. Nonetheless, despite being faced with these difficulties, this paper is the first, to our knowledge, to examine the definition and importance of secondary stressors following extreme events and disasters, and the first to offer a preliminary typology.

Additionally, on the basis of our review, we propose a pathway between extreme events and distress or mental disorders. Every disaster raises primary stressors, which may impact directly on people’s mental health. Continuing primary stressors may also become secondary stressors, or secondary stressors may occur directly from the extreme event. These secondary stressors also have the potential to cause distress and mental disorders and they are likely to intensify the effects of the primary stressors. Concurrent life events, which may be positive or negative, can also contribute to mental health problems or disorders that have already been produced by the primary or secondary stressors. Furthermore, the prevalence of mental disorders in the general population is such that a significant proportion of people who survive extreme events or disasters are likely to have had a mental disorder previously or have one at the time. These concurrent circumstances may also interact with the effects of primary and secondary stressors. This pathway is summarised by Figure 1.

**Suggested Pathway between an Extreme Event, Stressors and Distress or Mental Disorders.**
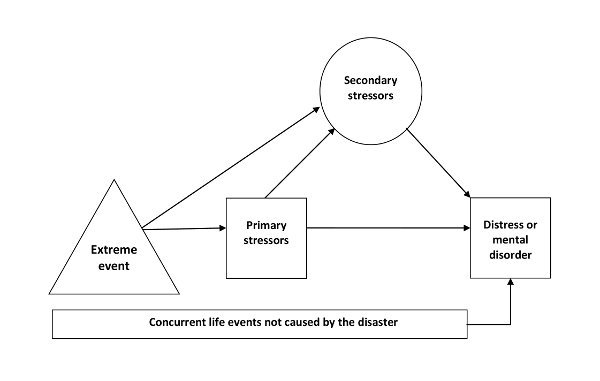
Note that not all of those people who are affected by the index extreme event and subsequent stressors develop mental health problems. The trajectories for recovery are not shown here.

**Limitations**

One limitation of our review is that we restricted our search to papers that had been published in 2010 and 2011. It is likely that we have missed other potential secondary stressors, such as disempowerment that may occur from stealing and looting after a disaster, impacts of sightseers and visitors to the site or area of an extreme event, failures in communication due to language and communication problems, or stigmatisation of a community. Reviews of papers published in the literature earlier than 2010 may reveal a wider range of secondary stressors.

Similarly, we do not consider in this paper which factors may be protective against or mitigate the development of distress or mental disorders, such as community support or financial resilience.

A key difficulty that we identified in our review was the lack of clear definitions and terminology for primary and secondary stressors, and other life events. The research we analysed measured secondary stressors using different mechanisms or tools, and the time interval following the event varied widely. This made it challenging to compare the different items that have been reported in the literature.

The absence of a consistently used terminology also often made it unclear to us which stressors were secondary stressors compared to those that were unresolved primary stressors, which, through their persistence, had become secondary stressors. The question also remains about whether or not these continuing primary stressors and people’s unmet needs should be called secondary stressors, or if they are better described as primary stressors that have become chronic. Also, it is not clear from much of the literature whether certain other life events were pre-existing and concurrent to the disaster, or caused or exacerbated by the index extreme event.

It is important to note that some secondary stressors are difficult to locate solely in one category of our typology or another. One example is loss of physical possessions or resources. Their loss can impact on a person’s mental health through grief at losing items of sentimental value or through the economic impact of having to purchase new items or experiencing problems replacing them due to delays in compensation or the rigid requirements of some insurance policies.

**Future research**

Our recommendations for future research are to develop and agree a definition of the core features of secondary stressors following extreme events and disasters, and to adopt more explicit definitions and descriptions of the secondary stressors that are studied. This would aid comparison of the effects found in different studies, as well as allowing clearer differentiation of which of the stressors studied are primary, and which are secondary. Secondary stressors should also be investigated as separate or unique items when examining their effects on mental disorders, rather than grouping together a number of stressors. This will enable better determination of the range and effects of secondary stressors that can occur following an event.

We think that future research should also consider the inter-relationships and pathways of interaction between secondary stressors, and between primary and secondary stressors. Examining the time line of when primary stressors change and become secondary stressors, or when distress changes and becomes a mental disorder, along with the possible reasons for these processes, would be helpful to policymakers, people who design and deliver services, and practitioners from a range of disciplines. These efforts will enable effective actions and interventions to be implemented early, to halt progression and limit the effects of disasters on mental health.

In the future, literature reviews should include papers published before 2010 in order to widen the typology of secondary stressors we have identified in this paper. Although identifying interventions that are effective in reducing the impact of secondary stressors was not an aim of our study, we note that none of the papers we included reported investigation of this aspect. Once a more substantive typology of secondary stressors is agreed, the next logical step would be to research which ones are amenable to action and what actions are shown to be effective. Future reviews could also investigate which are the secondary stressors that occur commonly following disasters in high-income as compared with low- and middle-income countries (see, e.g., 38). This information would help aid agencies to better focus their interventions.

## Conclusions

Our review identifies the importance of deepening our understanding of the diversity of people’s responses to disasters and understanding better why some people appear to be more resilient than others. Separating out the aspects of disasters that are inherent and potentially inevitable from those stressors that are avoidable and modifiable is essential if we are to mitigate or modify them with effective and timely responses. We should also understand the inter-relatedness of decisions and events: a decision made about one matter that might appear logical when it is made, but could rebound later and become a secondary stressor to people who already feel powerless in their changed circumstances.

It is important to construct a better understanding of the different types of secondary stressors with a view to developing a clearer picture of the common ones and, as we have done, develop a typology of secondary stressors. This will not only help to focus research design, but also shape investigation of effective interventions to reduce their impacts. Future research should focus on developing clearer criteria for what constitutes a secondary stressor and how to delineate them from primary stressors. Improved description of a particular secondary stressor that is studied will also encourage researchers to be more consistent in their approach to this topic, especially in terms of what is measured and how it is reported. More extensive reviews of the literature will also help to identify additional secondary stressors to add to our preliminary typology and thereby refine it. This will make it easier for researchers to identify which stressors are particularly important following extreme events and furthermore, facilitate assessment of the interactions between, and time scales for the impacts of, primary and secondary stressors.

After extreme events and disasters, the ultimate aim for mental health and public health professionals should be to reduce the mental health burden falling on people who are affected. Our identification of secondary stressors and categorisation of their various types is the beginning of this process. We hope that this paper contributes by highlighting the importance of the effects of secondary stressors after extreme events and disasters and that this leads to more effective and timely responses. We also hope that this paper encourages other researchers to pursue this topic towards its better understanding.

## Competing Interests

The authors have declared that no competing interests exist.

## Appendix A

### PRISMA Checklist

Download PDF
